# Potential benefits of an alternative haemoglobin deferral strategy evaluated in seven countries

**DOI:** 10.1111/vox.70131

**Published:** 2025-10-13

**Authors:** Amber Meulenbeld, Claire Styles, Glen Shuttleworth, Supun Manathunga, Hans van Remoortel, Lucile Malard, Tinus Brits, Ronel Swanevelder, Jose Antonio García‐Erce, Iris Garcia‐Martínez, Surendra Karki, Marijke Welvaert, W. Alton Russell, Mikko Arvas, Katja van den Hurk, Mart Pothast, Mart Janssen

**Affiliations:** ^1^ Donor Health, Department of Research Sanquin Blood Supply Foundation Amsterdam The Netherlands; ^2^ Department of Public and Occupational Health Amsterdam UMC Amsterdam The Netherlands; ^3^ Amsterdam Public Health Research Institute, Amsterdam UMC Amsterdam The Netherlands; ^4^ Pathology and Clinical Governance, Australian Red Cross Lifeblood Sydney Australia; ^5^ Research and Development, Australian Red Cross Lifeblood Sydney Australia; ^6^ Division of Experimental Medicine McGill University Montreal Canada; ^7^ Centre for Evidence‐Based Practice, Belgian Red Cross Brussels Belgium; ^8^ Department of Public Health and Primary Care KU Leuven Leuven Belgium; ^9^ Etablissement Français du Sang La Plaine Saint‐Denis France; ^10^ Business Intelligence, South African National Blood Service Johannesburg South Africa; ^11^ Banco de Sangre y Tejidos de Navarra Pamplona Spain; ^12^ Laboratori de Coagulopaties Congènites, Banc de Sang i Teixits (BST) Barcelona Spain; ^13^ Grup de Medicina Transfusional, Vall d'Hebron Institut de Recerca (VHIR), Universitat Autònoma de Barcelona (UAB) Barcelona Spain; ^14^ School of Population and Global Health, McGill University Montreal Canada; ^15^ Research and Development, Finnish Red Cross Blood Service Helsinki Finland; ^16^ Transfusion Technology Assessment, Department of Research Sanquin Blood Supply Foundation Amsterdam The Netherlands

**Keywords:** blood donation, donor deferral, Hb measurement, measurement variability

## Abstract

**Background and Objectives:**

On‐site donor deferral for low haemoglobin (Hb) levels poses significant challenges for blood establishments globally, leading to material wastage and consumption of valuable staff and donor time. Traditionally, donors are deferred based on a single visit's Hb measurement, without considering previous Hb levels and measurement variability. This study aims to quantify, in different settings, the potential impact of an alternative deferral algorithm based on historical mean Hb levels.

**Materials and Methods:**

We retrospectively reassessed donor eligibility in 20,430,816 donations and deferrals in Australia, Belgium, Finland, France, the Netherlands, South Africa and the United States using an algorithm that considers a repeat donor eligible as long as their historical mean Hb is above the deferral threshold and deviations from the mean are consistent with anticipated measurement variability. We quantified the potential impact of the alternative algorithm by calculating the change in donations and deferrals.

**Results:**

Across countries, the alternative algorithm may reduce low Hb deferrals between 30% and 70%. Additionally, in every country, a small proportion of current donors (~1%) donate who exhibit consistent low Hb levels. Balancing new deferrals and donations, the estimated net increase in donations across countries ranges between 0.7% and 3.3%.

**Conclusion:**

The alternative deferral algorithm based on mean Hb levels is a first step towards a more comprehensive assessment of Hb levels to determine donor eligibility. Further research is needed to refine the algorithm, to determine its long‐term impact, to improve the model with information related to iron stores and recovery and to address the impact on donor safety.

**Highlights**

- Deferring donors based on historical mean haemoglobin (Hb) levels may reduce Hb deferral rates in all participating blood establishments by up to 70%.
- The use of such an algorithm also identifies donors with consistently low Hb levels who are currently allowed to donate because of incidentally high Hb measurements.
- This is a first step towards a more comprehensive assessment of donor eligibility using historical Hb measurements, pending evidence on long‐term effects, further development of the algorithm and donor safety.

## INTRODUCTION

Ensuring a stable and sufficient blood supply is a persistent challenge for blood establishments, particularly due to donor recruitment and retention issues. One major factor affecting donor availability is the measurement of haemoglobin (Hb) to ensure donor safety. Blood establishments are typically deferring individuals whose Hb levels fall below established thresholds, such as the European Union's standards of 12.5 and 13.5 g/dL for females and males, respectively [1]. This is crucial to prevent adverse health effects in donors, such as iron deficiency and subsequent anaemia, which can result from donating blood with insufficient iron stores [2, 3]. However, the practice of on‐site deferral presents several challenges. Deferral can be demoralizing for donors, aggravating retention difficulties because it hampers return for donation, and it incurs material wastage and consumes valuable staff resources [4, 5, 6].

In pursuit of more effective blood donor management, various strategies have been explored. These include alternative measurement strategies that incorporate multiple iron parameters [7], prediction models for Hb levels [8], optimization of donation intervals [9] and the provision of iron supplements [10, 11]. Each of these strategies aims to minimize donor deferrals while maintaining donor health and a stable blood supply. Yet, a critical aspect that has received limited attention is the inherent variability in Hb measurements [12].

Blood establishments worldwide employ varying methods for Hb measurement to base their deferrals on. Their methods differ in devices, pre‐analytic procedures, blood draw techniques (venous vs. capillary), repeated measurements and timing of measurements (pre‐donation vs. post‐donation) [13]. All of these factors contribute to the inherent (pre‐)analytical variability in Hb measurements, referring to the variability in the measurement outcome as a result of the measurement method applied [14, 15, 16]. However, the measurement outcome is also affected by the *biological* variability, referring to the fluctuation in Hb levels as a result of internal physiological processes [17]. Both sources of variability may contribute to unnecessary donor deferrals or to donors donating with too low Hb levels [12]. Despite this, most donor deferral practices rely on single measurements or repeated measurements at a single visit without explicitly accounting for measurement variability, which might impact donor eligibility decisions.

Recently, an alternative donor deferral algorithm was proposed in which a donor's mean Hb level over the course of their career and the variability of Hb measurements are taken into account [12]. This approach mimics the standard laboratory practice principle of the control chart [18]. Our present study aims to quantify the potential of this alternative deferral algorithm in multiple blood establishments around the world in terms of changes in deferral rates and donations. Specifically, we seek to assess the reduction in potentially unnecessary deferrals and the identification of non‐eligible donors who are currently deemed eligible to donate.

## MATERIALS AND METHODS

### Alternative donor deferral algorithm

The deferral algorithm we applied to reassess deferrals was adapted from the algorithm described by Janssen [12]. In short, our algorithm evaluates a donor's historical mean Hb level and the measurement variability within the blood establishment to determine eligibility. A donor may be deferred for two reasons: (A) their historical mean Hb falls below the legal deferral threshold, or (B) an individual Hb measurement is classified as a low outlier with respect to the mean Hb.

**FIGURE 1:**
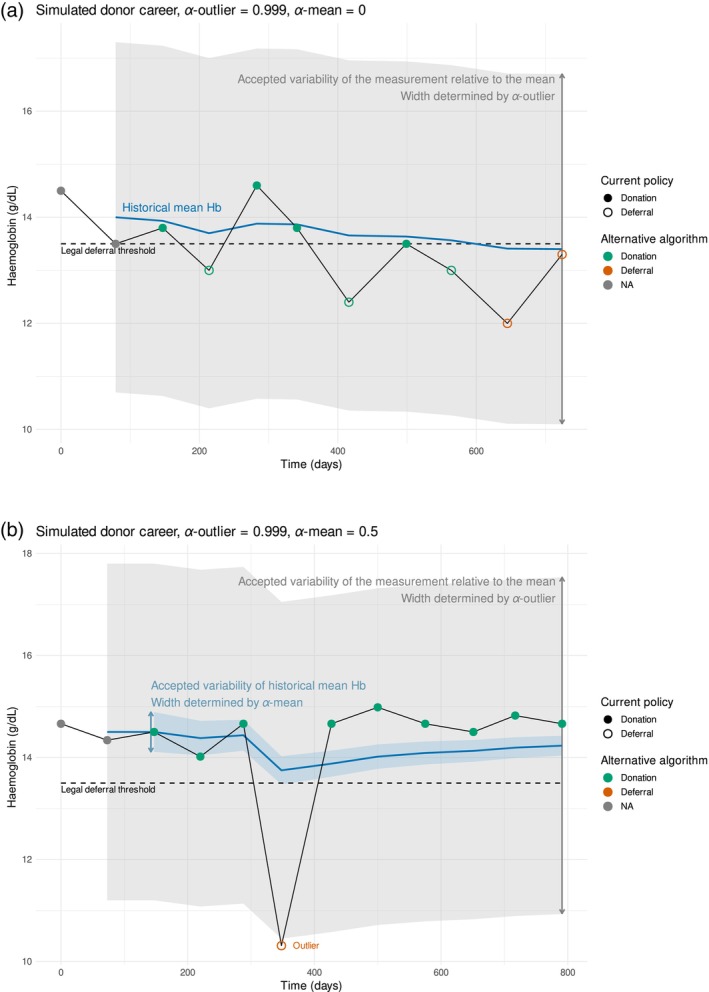
(a) Simulated donor career illustrating the current policy (shape of data points) versus the alternative algorithm (colour of data points). This shows an example of deferral because the historical mean haemoglobin (Hb) is below the legal deferral threshold (deferral reason A). There is no confidence interval around the mean because *α*‐mean is 0. (b) Simulated donor career illustrating the current policy (shape of data points) versus the alternative algorithm (colour of data points). This shows an example of a deferral because an observation is classified as a low outlier with respect to the mean Hb (deferral reason B). NA, not applicable.

The algorithm incorporates two key parameters. The first, *α*‐mean, defines the frequentist confidence interval for the mean with *α*‐mean × 100%, which indicates the range that would contain the true mean in repeated sampling. It can be set between −1 and 1, controlling the width of the confidence interval around the historical mean Hb based on the estimated standard deviation of the individual Hb measurement. In other words, this parameter controls how much uncertainty we allow in a donor's estimated mean Hb level based on their past donations. With a negative *α*‐mean, deferral occurs whenever the lower bound of the confidence interval falls below the deferral threshold. This helps in preventing donations from donors who may be at risk even if their average appears acceptable. When the *α*‐mean is zero, eligibility is based solely on the historical mean Hb, and donors are deferred whenever their mean Hb level is below the deferral threshold (Figure 1a). A positive *α*‐mean results in a more lenient approach: donors are deferred only when the upper bound of the confidence interval is below the threshold. In this case, even donors with a mean slightly below the threshold may still be considered eligible if their variability suggests that most of their actual Hb values would still be above the cutoff.

The second parameter, *α*‐outlier, addresses the variability of individual measurements. Even with a stable long‐term Hb level, occasional low readings can occur. The parameter *α*‐outlier sets the probability threshold used to determine whether a single Hb measurement should be classified as an outlier, essentially judging whether a low reading is likely to reflect a real drop or just the result of random variation. It can be set between 0 and 1, and donors are deferred only if their Hb measurement is identified as a low outlier (Figure 1b). An *α*‐outlier of 0.999 implies that only 1 in 1000 Hb measurements would be incorrectly classified as an outlier by chance. The process of deferral using the alternative algorithm is displayed in Figures S1 and S2.

### Data sources and selection

We extracted data of whole blood donation visits from the Australian Red Cross Lifeblood, Belgian Red Cross—Flanders in Belgium, the Finnish Red Cross Blood Service, the South African National Blood Service, Sanquin in the Netherlands and Vitalant in the United States. We only included donors for whom data was available from their first donation onwards and extracted the unique donor ID, donor sex, donation date and Hb level. Donation types other than whole blood donations were removed from the datasets. For the US site, we performed a sensitivity analysis excluding donors who performed double red cell donations in the past 12 months (Table S1). In Table 1, we provide an overview of the setting in the blood establishments from which we extracted data by listing key elements of their current Hb management policy. Some blood establishments perform repeated Hb measurements, which were handled as follows: In the Netherlands, up to three Hb measurements may be performed during the same visit if initial readings are low, but only the highest value is recorded and therefore used for this study. For the US site, if the initial Hb measurement is low, donors may return for retesting on a subsequent day. Both the initial and any follow‐up measurements are recorded, and for analysis we used the highest available Hb value. However, we performed sensitivity analyses with the minimum and average Hb values, which did not yield significantly different results.

**TABLE 1:** Haemoglobin measurement and deferral policies across participating blood establishments.

		Australia	Belgium (Flanders)^a^	Finland	France	The Netherlands	South Africa^a^	USA^a^
Invitation method		Appointment scheduling and walk‐ins	Appointment scheduling	Invitations, appointment scheduling and walk‐ins	Appointment scheduling and walk‐ins	Invitations	Invitations	Invitations and walk‐in
Minimum donation interval/maximum number of donations	Males	84 days	60 days and 4 donations/year	56 days and 6 donations/year	56 days and 6 donations/year	56 days and 5 donations/year	56 days (above age 65: 84 days)	56 days
	Females	84 days	60 days and 4 donations/year	56 days and 4 donations/year	56 days and 4 donations/year	122 days and 3 donations/year	56 days (above age 65: 84 days)	56 days
Sample origin for Hb measurement		Capillary	Venous, capillary if previous post‐donation venous measurement was below the eligibility threshold	Capillary	Venous, capillary if: new donors previous post‐donation venous measurement was below 12.5 g/dL for women and 13.5 g/dL for men clinical signs of anaemia >2 years since last donation	Capillary	Capillary	Capillary
Hb measurement method		Fresenius Kabi Compolab TM	Fresenius Kabi Compolab TM (capillary sample), Sysmex XN1000 (venous sample)	HemoCue 201	HemoCue 201 (capillary sample), Sysmex XN9100 (venous sample)	HemoCue 201	HemoCue 301	HemoCue 301
Hb measurement timing		Pre‐donation	Post‐donation, capillary pre‐donation	Pre‐donation	Post‐donation, capillary pre‐donation	Pre‐donation	Pre‐donation	Pre‐donation
Repeated Hb measurement		No	No	No	No	Yes, up to three times, highest value is recorded	No	Yes, up to two times, all values are recorded
Deferral threshold (g/dL)	Males	13.0	13.5	13.0	13.0	13.5	13.5	13.0
	Females	12.0	12.5	12.0	12.0	12.5	12.5	12.5
Deferral period		6 months	3 months	3 months	6 months	3 months	3 months	Until next day

Abbreviation: Hb, haemoglobin.

^a^ Belgian Red Cross—Flanders, South African National Blood Service and Vitalant are not national organizations and the described policy is not necessarily applicable in the whole country.

### Data analysis

First, for each country separately, Hb measurement variability was estimated using all available data from that country by calculating the standard deviation of the difference between subsequent Hb measurements and dividing by √2. This gives an upper limit for the standard deviation of the measurement variability (including biological variation). We assessed that correcting this estimate for donor recovery after donation (which shows a linear increase when plotted on a log(time) scale) had a very small effect, so this recovery effect was omitted.

Next, using the estimated measurement variability and deferral thresholds in each country, we reassessed donor eligibility of donations and deferrals in each blood establishment using the alternative deferral algorithm. Because the algorithm focuses on Hb deferrals, we assume a deferral took place if the donor's Hb was below the threshold outlined in Table 1. Other types of deferrals are not taken into account.

We varied *α*‐mean between −0.999 and 0.999 and used *α*‐outlier at 0.99, 0.999 and 0.9999. With these parameter configurations, we calculated the deferral rate in the dataset under the country's current policy and the deferral rate with the new algorithm. In addition, we calculated the proportion of ineligible donations, that is, donations that took place but would have been deferred with the alternative algorithm. As the algorithm prompts a change in deferrals and classifies some current donations as ineligible, we also calculate the total change in donations. Lastly, to assess the impact of the alternative strategy on Hb levels, we calculated the mean Hb level of the historical mean Hb of newly eligible donations under the alternative deferral algorithm. The algorithm cannot be applied to the first two donations, as the confidence interval of the historical mean Hb cannot be established before the third donation (Figure 1b). Therefore, the first two donations are disregarded from the calculations.

Analyses were performed locally by each blood establishment using R language for statistical computing [19]. The R scripts used for analysis and production of figures are available on GitHub (https://github.com/Sanquin/SanguinStats---Donor-Deferral-project).

## RESULTS

The mean Hb levels and standard deviations differ between countries and measurement methods (Table 2). Mean Hb levels for males were highest in South Africa (15.66 g/dL) and lowest in Australia (14.84 g/dL). For females, mean Hb levels were highest in Finland (13.89 g/dL) and lowest in France (13.31 g/dL). In all countries, the Hb levels of female donors are around 10% lower than those of male donors. Belgium's measurement showed the lowest standard deviation (0.49 g/dL for males, 0.52 g/dL for females), followed by France, which is not surprising as these are the only countries in our sample that perform Hb measurements with a haematology analyser using venous blood samples (Table 1). The standard deviations for capillary blood measurements are approximately 0.70 g/dL. However, in countries that use the HemoCue 301, they are slightly higher—South Africa (0.83 g/dL for females and 0.84 g/dL for males) and the United States (0.79 g/dL for females and 0.85 g/dL for males) (Table 2).

**TABLE 2:** Mean haemoglobin levels and mean standard deviation of the haemoglobin measurement per blood establishment.

	Australia	Belgium	Finland	France	The Netherlands	South Africa	USA
Males							
Mean Hb (g/dL)	14.84	15.19	15.58	15.13	15.06	15.66	15.55
Standard deviation (g/dL)	0.71	0.49	0.73	0.53	0.70	0.84	0.85
Females							
Mean Hb (g/dL)	13.37	13.65	13.89	13.31	13.54	13.70	13.68
Standard deviation (g/dL)	0.67	0.52	0.73	0.62	0.67	0.83	0.79

Abbreviation: Hb, haemoglobin.

Table 3 summarizes the results of applying the alternative deferral algorithm with *α*‐mean of 0 and *α*‐outlier of 0.999, compared to each country's current strategy. Results with varying parameter configurations are displayed in Figures S3–S9. The analysis included 20,436,791 donations and deferrals from 2,965,600 donors across seven countries, collected from 1999 to 2025. The application of the alternative algorithm led to a potential reduction in deferrals between 37% and 70%. The highest reduction in deferral rate was observed in Australia (69.7%), while the smallest reduction in deferral rate, in the United States (36.8%), was still substantial. The overall mean of individual donors' historical mean Hb at newly eligible donations with the alternative algorithm ranges from 13.5 to 14.4 g/dL in males and from 12.6 to 13.1 g/dL in females.

**TABLE 3:** Deferral rates with current and alternative deferral strategies in different countries.

		Australia	Belgium^a^	Finland	France^a^	The Netherlands	South Africa	USA
Date range		May 2014–May 2024	Jun 2015–Jan 2025	Dec 1999–Dec 2019	Jan 2015–Dec 2024	Jan 2009–Dec 2023	Jan 2019–Feb 2025	Jan 2017–Oct 2022
Donors		420,708	123,004	140,435	1,018,235	306,320	436,056	520,842
Donation attempts analysed		3,364,513	684,926	1,113,715	6,158,873	2,597,503	2,970,291	3,546,970
Deferral rate with current strategy (%)		1.42	4.29	4.30	5.05	5.22	4.48	8.10
Deferral rate with alternative algorithm (%)		0.43	1.70	1.59	2.27	2.49	1.92	5.12
Change in deferrals (%)		−69.7	−60.4	−63.0	−55.1	−52.3	−57.1	−36.8
Donations that are currently accepted but would have been deferred under the alternative algorithm (%)		0.23	0.60	0.60	0.71	1.05	0.72	1.97
Change in donations (%)^b^		+1.0	+2.71	+2.84	+2.93	+2.88	+2.68	+3.25
Mean historical mean Hb of donations newly eligible under the alternative strategy (g/dL)	Males	13.8	14.0	14.4	13.7	14.2	13.9	13.7
	Females	12.6	12.9	13.1	12.6	13.0	12.7	13.0

*Note*: The analysis was performed with an *α*‐mean of 0 and *α*‐outlier of 0.999.

Abbreviation: Hb, haemoglobin.

^a^ Note that as Belgium and France perform a post‐donation Hb test, deferral rates here are post‐donation deferrals, whereas for all other countries, deferral rates refer to on‐site deferrals. In Belgium and France, deferred donors are subject to a pre‐donation re‐entry Hb test. These tests are not considered in this assessment.

^b^ Note that the % change in donations differs from the change in deferral rates between the current and alternative strategies (presented in earlier rows) because the denominators used for calculating these percentages are different. The difference between these proportions is per country in the order of the deferral rate.

Despite the reduction in deferral rates, a small number of donations that are considered eligible under the current policy would not have been allowed when applying the alternative algorithm because the donors exhibit consistently low Hb levels. This percentage ranges from 0.23% in Australia to 1.97% in the United States. By balancing new deferrals and new donations in every country, all countries see a net increase in donations compared to the current strategy, ranging from 1.0% in Australia to 3.25% in the United States. In the US dataset, 6.72% of donors had given a double red cell donation in the preceding year. A sensitivity analysis showed negligible impact on deferral reductions (Table S1).

## DISCUSSION

Our study demonstrates that an alternative deferral algorithm based on a historical mean Hb to account for measurement variability could substantially reduce on‐site deferral rates across various blood establishments [12]. The algorithm increased the proportion of visits at which a donation could be collected by 1.0%–3.25% across countries, highlighting the significant impact of addressing measurement variability and the potential to improve donor yield and operational efficiency across blood services worldwide. While the alternative algorithm potentially decreases unnecessary deferrals in all countries, it also identifies a small proportion of current donors (ranging from 0.23% to 1.97%) who have consistently low Hb levels and should therefore be deferred (temporarily or maybe even permanently) from donation.

The alternative deferral algorithm presents several advantages. First, it allows for natural fluctuations in Hb levels while protecting donors from donating with anomalously low Hb levels. This is particularly relevant because capillary Hb measurements can be affected by pre‐analytical factors such as hydration status, hand temperature and interstitial fluid, which may lead to unnecessary deferrals under strict single‐measurement criteria [20]. However, even in countries using haematology analysers for post‐donation Hb measurements, the variability is still substantial, and the alternative algorithm thus achieves similar reductions in deferral rates.

By considering historical Hb trends, the algorithm helps in ensuring that donors are not deferred because of momentary fluctuations while still identifying those with persistently low Hb levels who may be at risk of iron deficiency and anaemia. Our findings show that approximately 1% of current donors exhibit consistently low Hb levels, reinforcing the need to account for individual haematological patterns in donor management. This aligns with studies introducing the concept of an individual haematological setpoint that is stable over an extended period [21]. This research indicates that each individual maintains a relatively consistent Hb level, which further supports the importance of longitudinal Hb monitoring in donor screening. Another benefit of reducing on‐site deferrals is the potential for improved donor retention. Studies have shown that deferrals are a strong predictor of non‐return and can be demoralizing, often leading to decreased donor return rates [4, 5, 6, 7, 22]. However, by lowering deferral rates, the algorithm may help mitigate this effect, provided donor health is not compromised. Notably, the immediate impact of the alternative algorithm on donor retention may be less pronounced in some countries, like the United States, where donors can return as soon as the next day. Likewise, in Belgium and France, where deferrals occur post donation rather than on site, the primary benefit is the ability to invite donors back earlier, and reducing deferrals does not improve staff efficiency or material wastage.

Although the proposed algorithm offers clear advantages, it also has limitations that warrant careful consideration. Since it relies on historical data to establish a reference value, at present it does not provide any guidance on how to handle the first few donations of new donors, like the first two donations in Figure 1a,b. Additionally, the proposed approach, based solely on the premise that a donor is deferred in case the mean Hb level is demonstrably below the threshold, may be too permissive. Moreover, we lack insight into the recovery of donors who are currently deferred, as well as data on their iron stores, which are crucial for Hb replenishment. It may also be that some momentary deviations could in fact reflect actual drops in Hb levels, for example, due to gastrointestinal disease. Further research is needed to determine what levels of low Hb can be considered acceptable without posing a risk to donor health and whether additional iron measurements, such as ferritin, could enhance donor safety while using this algorithm. The current algorithm might be improved by incorporating information on the recovery of Hb levels and iron stores after blood donation and may be tailored to individual donor characteristics like age, sex, race and donation history.

Given these considerations, blood establishments would be advised to tailor the algorithm to their specific contexts, capabilities and operational practices while ensuring compliance with prevailing regulations. In Europe, deviations from the Hb deferral threshold for single measurements are permitted for substantiated reasons, but this flexibility may not be globally accepted. The algorithm's key parameters can be adjusted based on the level of risk deemed acceptable in a given local context. These include the values for *α*‐mean and *α*‐outlier, as well as the number of past donations factored into the historical mean—whether all available data, as presented here, or only a limited number of most recent donations. A shorter history could, for example, also be considered for the historical mean Hb of postmenopausal women to accommodate their physiological change. Finally, the algorithm's policy implications can be tailored. While this study focuses on deferral, other interventions—such as confirmation tests, permanent deferral or alternative measures—could also be considered as outcomes. Additionally, although the donor might not be deferred under the alternative algorithm, it might be advisable to refer them to a healthcare provider if Hb levels are under public health thresholds.

Although the alternative deferral algorithm shows promise in reducing deferral rates and improving donor management, its feasibility and adoption depend on further refinements, deeper insights into biological variability in blood donors and regulatory acceptance of a fully operationalized strategy. A more nuanced discussion of the decision parameters will be essential to ensure a deferral strategy that reliably determines an acceptable donor eligibility outcome based on both current and historical Hb levels. Successfully implementing such a strategy will require close collaboration among researchers, blood establishments and regulators to address potential concerns and ensure donor safety.

The alternative deferral algorithm presented in this paper is a promising step towards a more comprehensive donor eligibility assessment. Once fully developed and validated, it could potentially reduce a substantial number of unnecessary deferrals, benefiting both blood establishments and donors. Future research should focus on prospective monitoring of donors' Hb levels regardless of deferral status, refining the algorithm decision parameters and assessing long‐term effects of such algorithms on donor health and blood supply sufficiency. Advancing donor deferral algorithms will require striking the right balance between optimizing donor health, minimizing risks and ensuring an adequate and (cost)efficient blood supply.

## CONFLICT OF INTEREST STATEMENT

All authors except S.M. and W.A.R. are employed by a national or regional blood service, responsible for (part of) the blood supply in their country.

## Supporting information


**Data S1.** Supporting information.

## Data Availability

The data that support the findings of this study are available on request from the corresponding author. The data are not publicly available due to privacy or ethical restrictions.

## References

[1] EDQM . Guide to the preparation, use and quality assurance of blood components. Strasbourg: European Directorate for the Quality of Medicines and HealthCare of the Council of Europe; 2023.

[2] Prinsze FJ , de Groot R , Timmer TC , Zalpuri S , van den Hurk K . Donation‐induced iron depletion is significantly associated with low hemoglobin at subsequent donations. Transfusion. 2021;61:3344–3352. 34596892 10.1111/trf.16688

[3] Boulton F . Managing donors and iron deficiency. Vox Sang. 2004;87:22–24. 15209872 10.1111/j.1741-6892.2004.00448.x

[4] Spekman MLC , Tilburg TG , Merz E . Do deferred donors continue their donations? A large‐scale register study on whole blood donor return in the Netherlands. Transfusion. 2019;59:3657–3665. 31621923 10.1111/trf.15551 PMC6916571

[5] Davison TE , Masser BM , Gemelli CN . Deferred and deterred: a review of literature on the impact of deferrals on blood donors. ISBT Sci Ser. 2020;15:3–10.

[6] Jagirdar H , Nwobi NH , Swanevelder R , Cockeran R , Bruhn R , Kaidarova Z , et al. Blood donor return behavior in South Africa and the United States before and during the COVID‐19 pandemic. Transfusion. 2024;64:1492–1502. 38940011 10.1111/trf.17934

[7] Meulenbeld A , Ramondt S , Sweegers MG , Quee FA , Prinsze FJ , Hoogendijk EO , et al. Effectiveness of ferritin‐guided donation intervals in whole‐blood donors in the Netherlands (FIND'EM): a stepped‐wedge cluster‐randomised trial. Lancet. 2024;404:31–43. 38880108 10.1016/S0140-6736(24)01085-7

[8] Vinkenoog M , Toivonen J , Brits T , de Clippel D , Compernolle V , Karki S , et al. An international comparison of haemoglobin deferral prediction models for blood banking. Vox Sang. 2023;118:430–439. 36924102 10.1111/vox.13426

[9] Toivonen J , Koski Y , Turkulainen E , Prinsze F , della Briotta Parolo P , Heinonen M , et al. Prediction and impact of personalized donation intervals. Vox Sang. 2022;117:504–512. 34825380 10.1111/vox.13223 PMC9299493

[10] Karregat J , Sweegers MG , Quee FA , Weekamp HH , Swinkels DW , Novotny VMJ , et al. Ferritin‐guided iron supplementation in whole blood donors: optimal dosage, donor response, return and efficacy (FORTE)‐a randomised controlled trial protocol. BMJ Open. 2022;12:e056316. 10.1136/bmjopen-2021-056316 PMC8915278 35264362

[11] Pasricha SR , Marks DC , Salvin H , Brama T , Keller AJ , Pink J , et al. Postdonation iron replacement for maintaining iron stores in female whole blood donors in routine donor practice: results of two feasibility studies in Australia. Transfusion. 2017;57:1922–1929. 28518220 10.1111/trf.14173

[12] Janssen MP . Why the majority of on‐site repeat donor deferrals are completely unwarranted…. Transfusion. 2022;62:2068–2075. 36053780 10.1111/trf.17085

[13] Chaudhary R , Dubey A , Sonker A . Techniques used for the screening of hemoglobin levels in blood donors: current insights and future directions. J Blood Med. 2017;8:75–88. 28740442 10.2147/JBM.S103788 PMC5503668

[14] Baart AM , de Kort WL , van den Hurk K , Pasker‐de Jong PC . Hemoglobin assessment: precision and practicability evaluated in the Netherlands – the HAPPEN study. Transfusion. 2016;56:1984–1993. 26968697 10.1111/trf.13546

[15] Ziemann M , Lizardo B , Geusendam G , Schlenke P . Reliability of capillary hemoglobin screening under routine conditions. Transfusion. 2011;51:2714–2719. 21599674 10.1111/j.1537-2995.2011.03183.x

[16] Bond MM , Richards‐Kortum RR . Drop‐to‐drop variation in the cellular components of fingerprick blood: implications for point‐of‐care diagnostic development. Am J Clin Pathol. 2015;144:885–894. 26572995 10.1309/AJCP1L7DKMPCHPEH

[17] Badrick T . Biological variation: understanding why it is so important? Pract Lab Med. 2021;23:e00199. 33490349 10.1016/j.plabm.2020.e00199 PMC7809190

[18] Bradford PG , Miranti PJ . Information in an industrial culture: Walter A. Shewhart and the evolution of the control chart, 1917–1954. Inf Cult. 2019;54:179–219.

[19] R Core Team . R: a language and environment for statistical computing. 2020. Available from: https://www.R‐project.org/. Last accessed 30 Jul 2025.

[20] Whitehead RD , Mei Z , Mapango C , Jefferds MED . Methods and analyzers for hemoglobin measurement in clinical laboratories and field settings. Ann N Y Acad Sci. 2019;1450:147–171. 31162693 10.1111/nyas.14124 PMC6709845

[21] Foy BH , Petherbridge R , Roth MT , Zhang C , De Souza DC , Mow C , et al. Haematological setpoints are a stable and patient‐specific deep phenotype. Nature. 2025;637:430–438. 39663453 10.1038/s41586-024-08264-5 PMC12085991

[22] Custer B , Schlumpf KS , Wright D , Simon TL , Wilkinson S , Ness PM , et al. Donor return after temporary deferral. Transfusion. 2011;51:1188–1196. 21155833 10.1111/j.1537-2995.2010.02989.x PMC3536538

