# Supplementary material for: Potential benefits of an alternative haemoglobin deferral strategy evaluated in seven countries
Source: Vox Sang. 2025 Oct 13;121(1):26–34. doi: 10.1111/vox.70131 (PMC12803770; doi:10.1111/vox.70131)
Supplement: Supplementary file 1 — Data S1. Supporting information. [file VOX-121-26-s001.docx]

Supplementary material to the paper ‘A Potential Alternative Hemoglobin Deferral Strategy Evaluated in Seven Countries’

Contents

[**Supplementary Figure 1.** Process of deferral with alternative deferral algorithm for blood establishments with a pre-donation Hb measurement policy 2](#_Toc204867874)

[**Supplementary Figure 2.** Process of deferral with alternative deferral algorithm for blood establishments with a post-donation Hb measurement policy 3](#_Toc204867875)

[**Supplementary Figure 3.** Deferral rate with alternative algorithm 4](#_Toc204867876)

[**Supplementary Figure 4.** Reduction in deferral rate 5](#_Toc204867877)

[**Supplementary Figure 5.** Increase in number of donations compared to maximum increase 6](#_Toc204867878)

[**Supplementary Figure 6.** Percentage of ineligible donations (donations that were allowed with the current algorithm and deferred with the alternative algorithm) 7](#_Toc204867879)

[**Supplementary Figure 7.** Mean of mean Hb of new donors with alternative strategy 8](#_Toc204867880)

[**Supplementary Figure 8.** Percentage of mean deferrals 9](#_Toc204867881)

[**Supplementary Figure 9.** Percentage of outlier deferrals 10](#_Toc204867882)

[**Supplementary Table 1.** Sensitivity analysis results 11](#_Toc204867883)


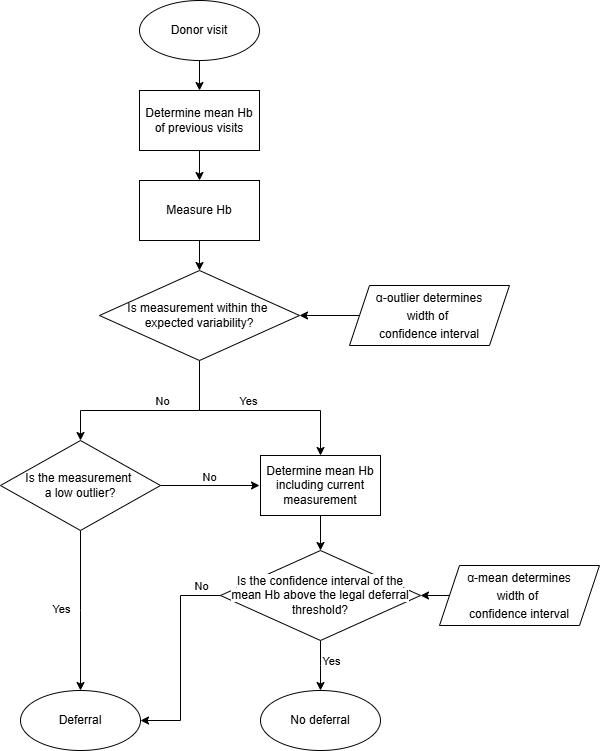


# **Supplementary Figure 1.** Process of deferral with alternative deferral algorithm for blood establishments with a pre-donation Hb measurement policy


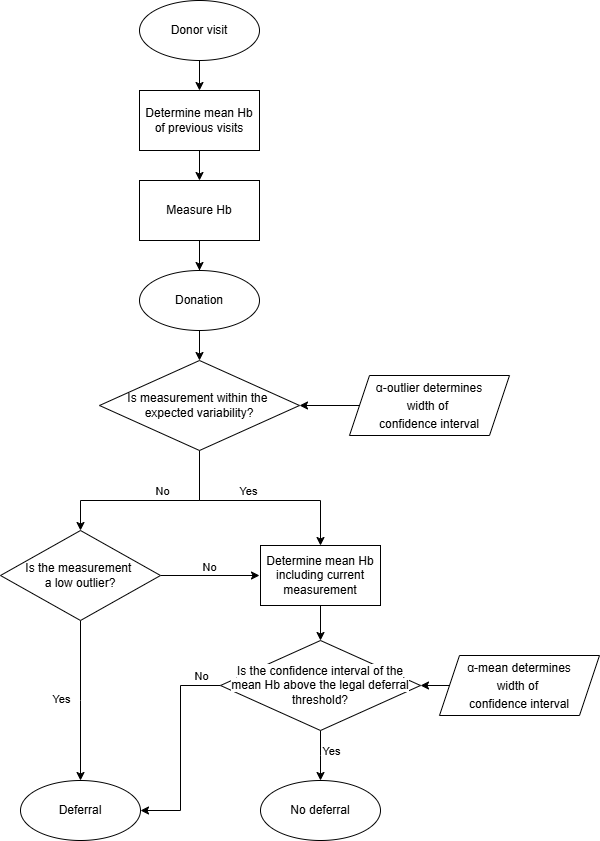


# **Supplementary Figure 2.** Process of deferral with alternative deferral algorithm for blood establishments with a post-donation Hb measurement policy


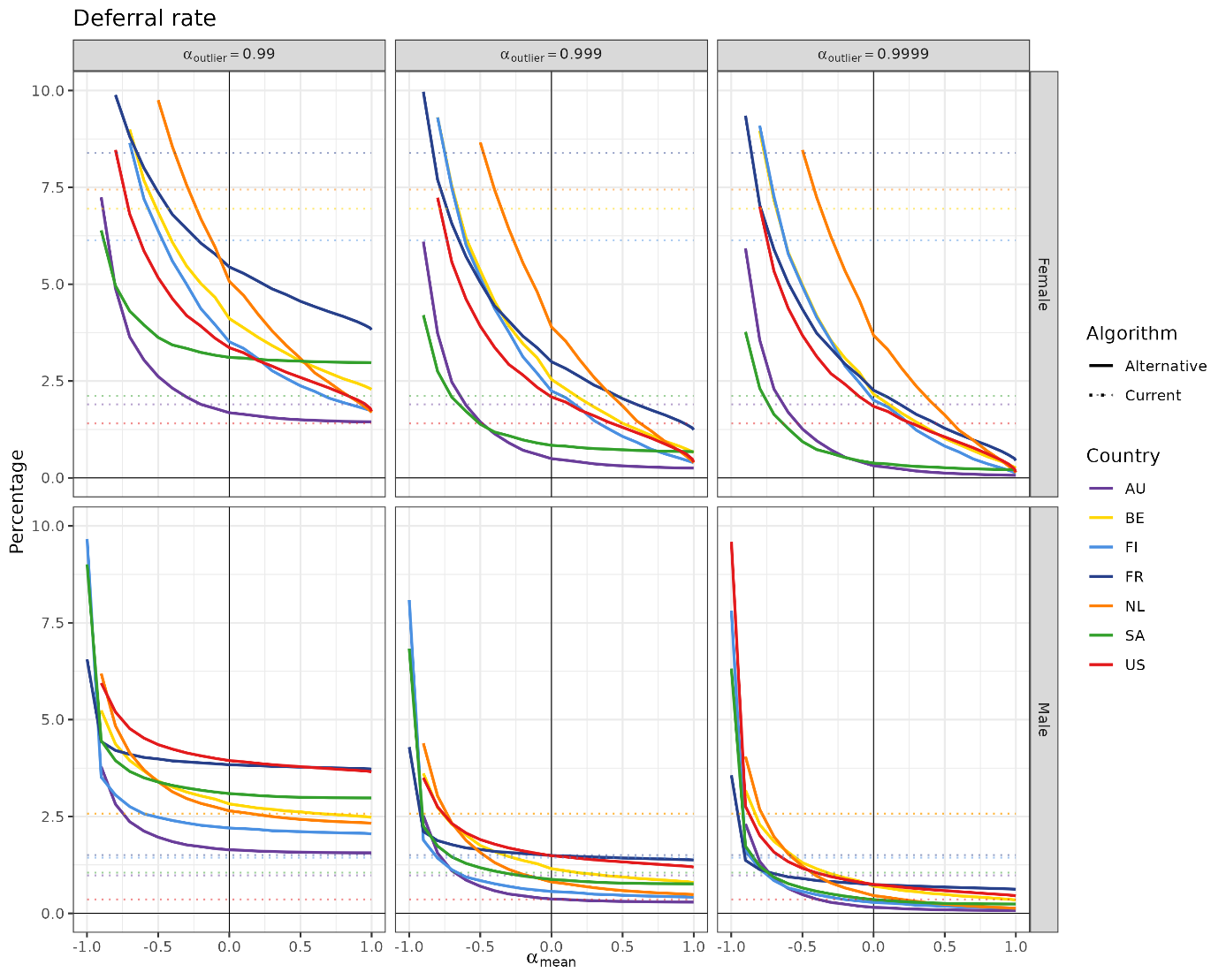


# **Supplementary Figure 3.** Deferral rate with alternative algorithm

These panels show the deferral rates as a function of α-mean for different α-outlier settings (0.99, 0.999, and 0.9999, from left to right columns). Separate curves are shown for females (top row) and males (bottom row), and for each participating country (colored lines; see legend). Solid lines represent deferral rates under the alternative algorithm across varying α-mean values; dotted horizontal lines represent the observed deferral rates under the current deferral policy for each country.

Deferral rates decrease as α-mean increases, reflecting more lenient deferral thresholds when more weight is given to the historical average Hb levels. Across all countries, the alternative algorithm at higher α-outlier values (i.e., more conservative outlier detection) would result in a substantial reduction in deferral rates compared to current practice, particularly for women.


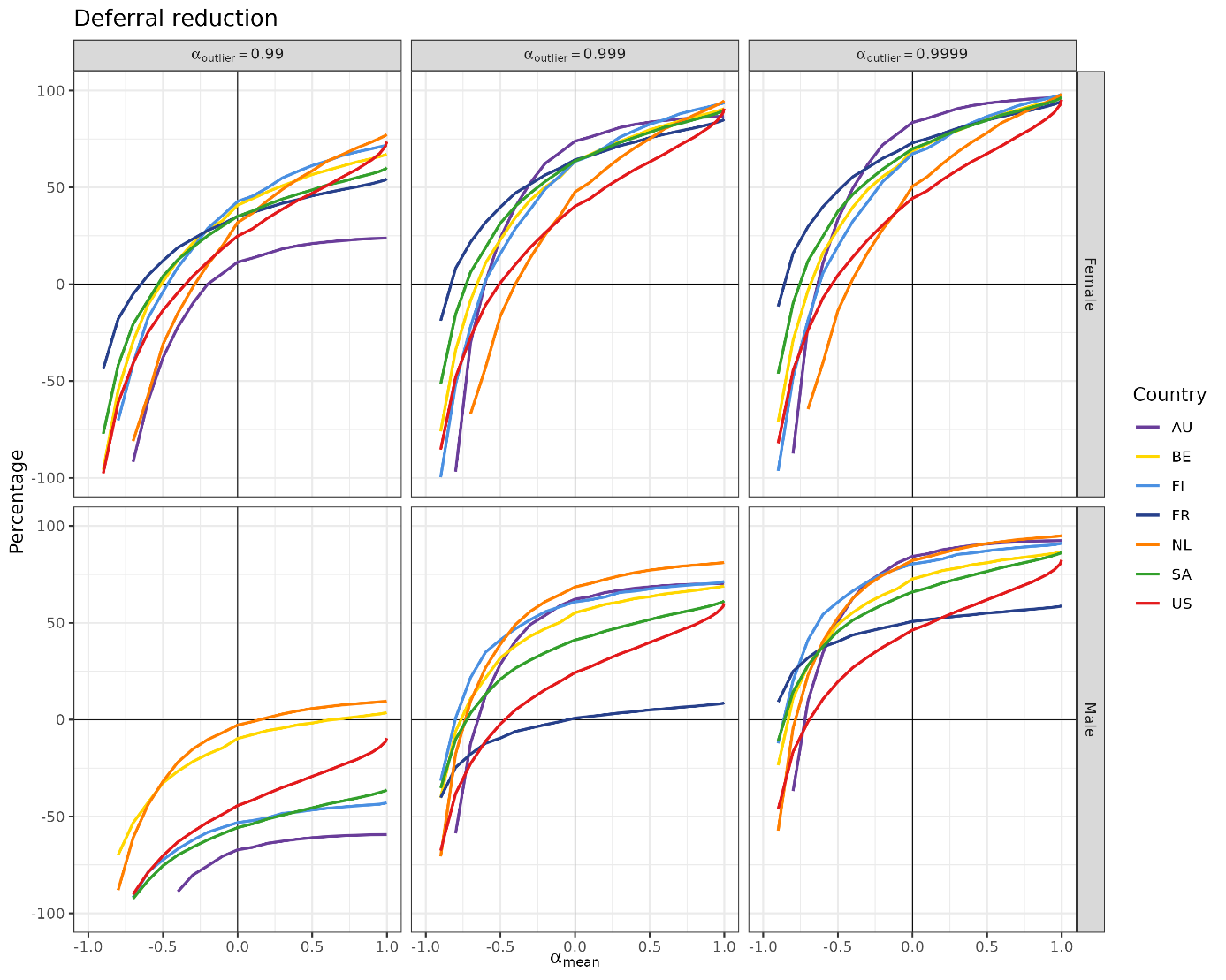


**Supplementary Figure 4.** Reduction in deferral rate

These panels show the percentage reduction in deferral rates for the alternative algorithm compared to current practice, across a range of α-mean values (x-axis), for each α-outlier setting (panel columns). Positive values indicate a reduction in deferrals compared to current practice; negative values indicate an increase. Separate results are presented for females (top row) and males (bottom row), with country-specific curves shown in color (see legend).

Deferral reductions increase with higher α-mean values, reflecting the more lenient use of historical Hb levels when less conservative confidence margins are applied. The effect of α-outlier is also evident: stricter outlier detection results in larger deferral reductions, particularly in female donors where deferral rates are generally higher under current practice. In male donors, setting α-outlier at 0.99 almost never achieves a reduction in deferral rate.

At high α-mean and α-outlier values, deferral reductions exceed 50% for most countries for both sexes, indicating substantial potential to reduce deferrals while still taking historical Hb variability into account.


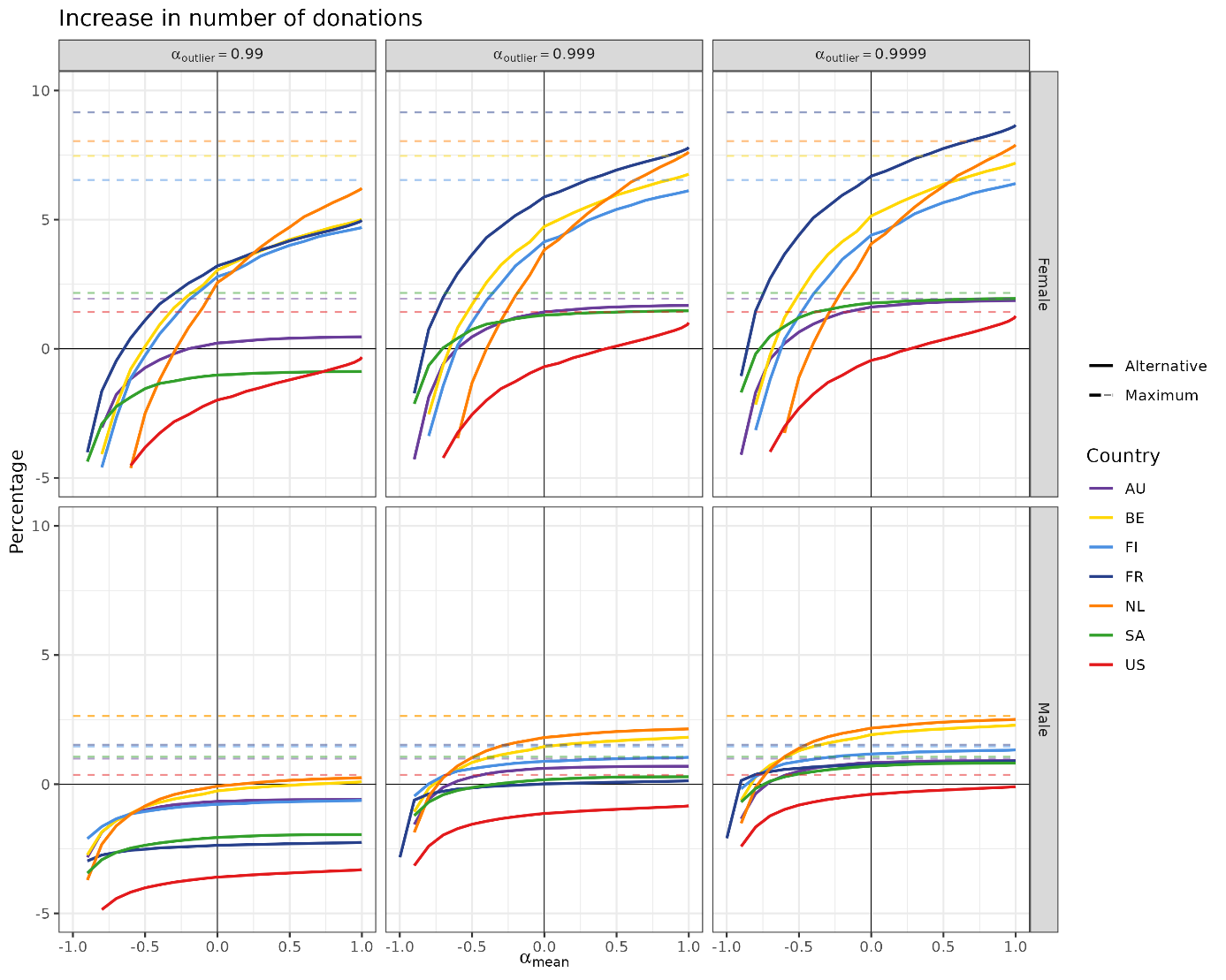


# **Supplementary Figure 5.** Increase in number of donations compared to maximum increase

These panels show the percentage change in total number of donations under the alternative algorithm, compared to current practice, across varying α-mean values (x-axis) and α-outlier settings (panel columns). Positive values indicate an increase in the number of successful donations; negative values indicate a decrease. Results are stratified by sex (panel rows), with country-specific curves shown in color (see legend). Solid lines represent the alternative algorithm, while the dashed horizontal lines indicate the theoretical maximum gain in donations if all donors who were deferred under the current algorithm would instead have been able to donate (i.e., the maximum possible gain assuming full eligibility).

As α-mean increases, allowing the more lenient use of historical Hb levels, the number of successful donations increases. The effect is stronger in female donors, who generally experience more deferrals under current policies. Similarly, stricter α-outlier thresholds (i.e., higher α-outlier values) lead to larger increases in donations, as fewer borderline low measurements are classified as outliers.


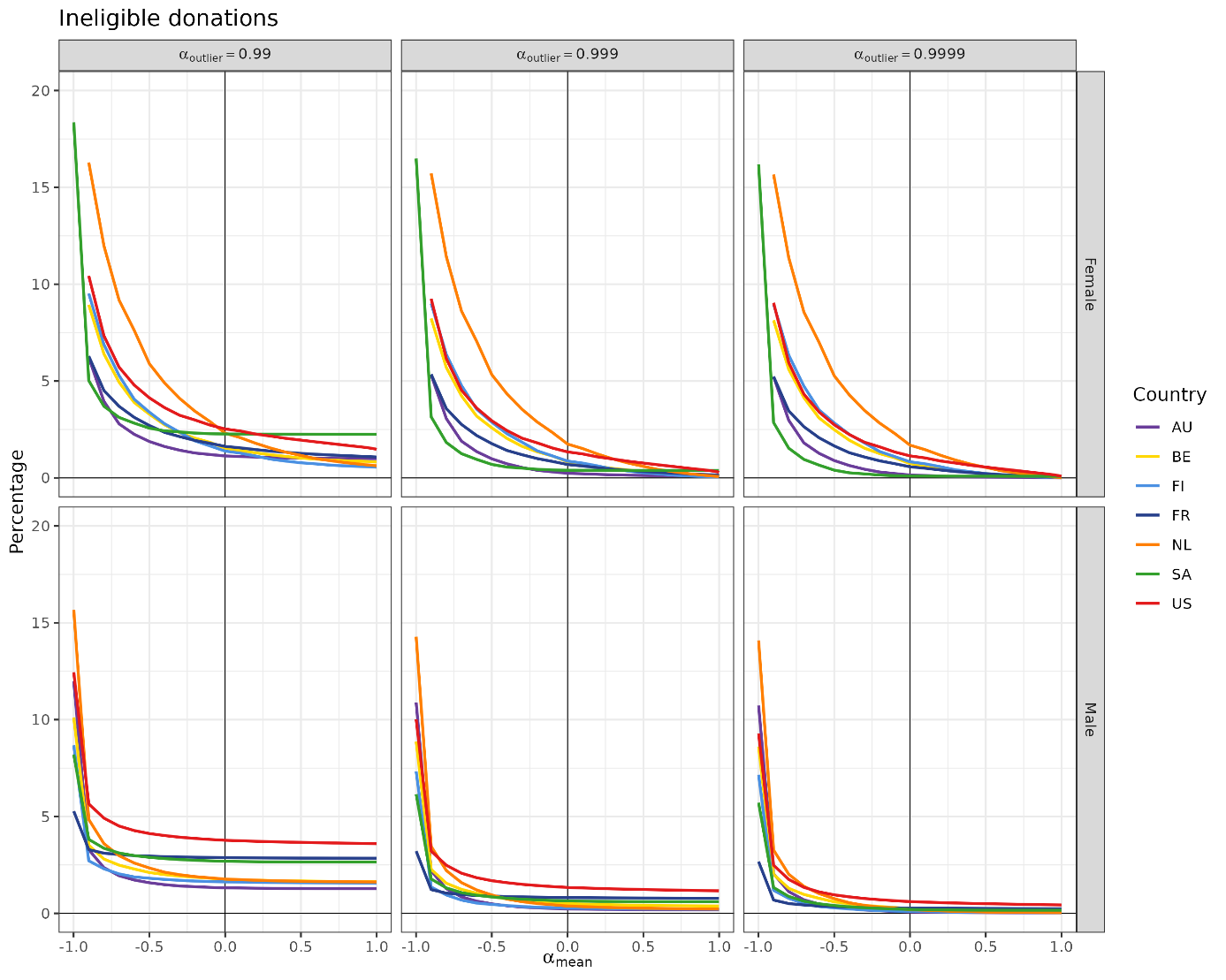


# **Supplementary Figure 6.** Percentage of ineligible donations (donations that were allowed with the current algorithm and deferred with the alternative algorithm)

This figure shows the percentage of donations that would be considered ineligible under the alternative algorithm, i.e. donations that were allowed under current deferral practice but would have been deferred using the alternative approach. Results are presented across a range of α-mean values (x-axis), for each α-outlier setting (panel columns), and stratified by sex (panel rows). Country-specific curves are shown in color according to the legend.

As α-mean increases, the proportion of ineligible donations decreases across all countries and both sexes because more donations will be allowed. At more conservative α-mean values (particularly for α-mean < 0), the algorithm is more restrictive, resulting in higher rates of ineligible donations. As with prior figures, the overall proportion of ineligible donations is higher in females than in males due to the higher overall deferral rates in female donors.


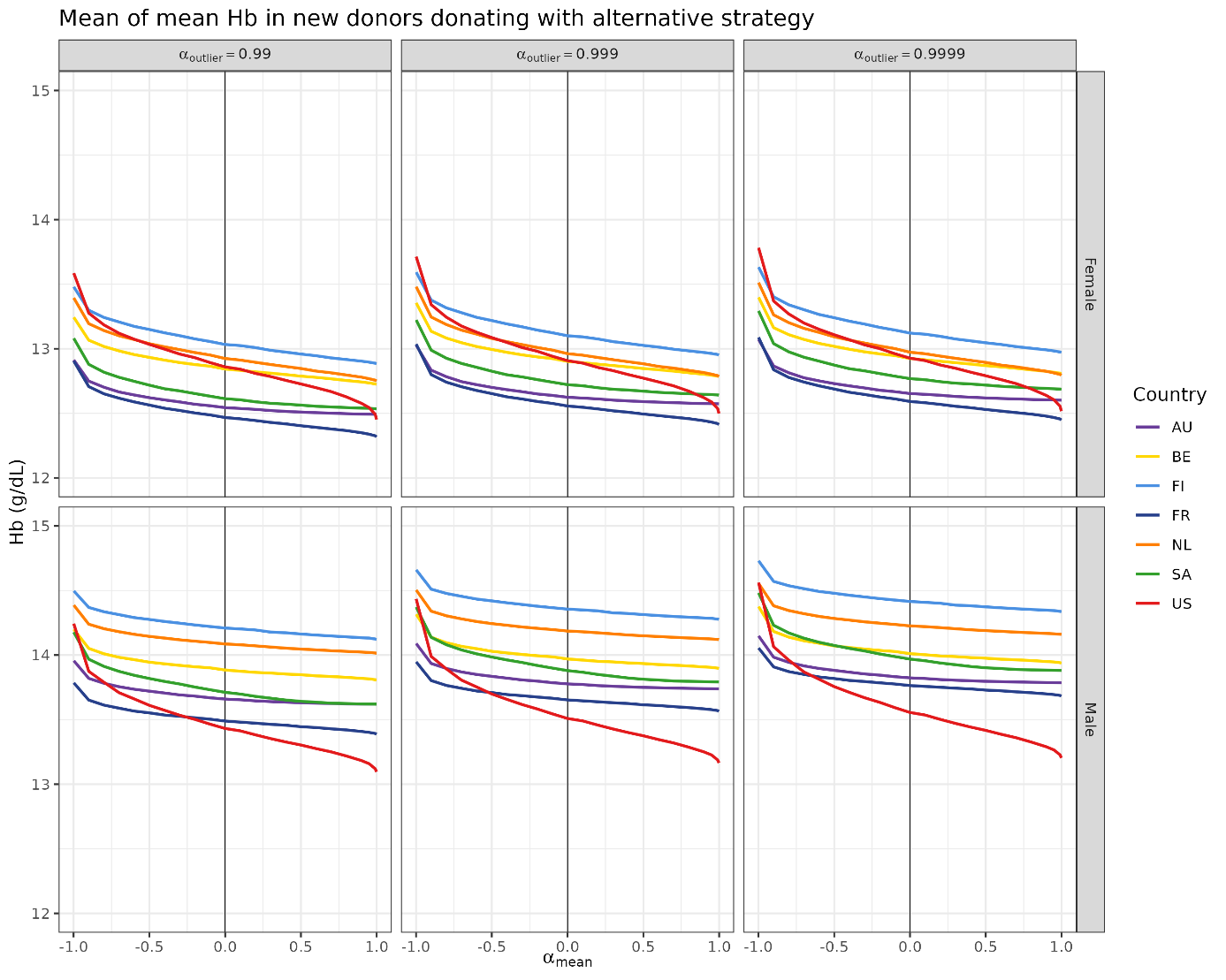


# **Supplementary Figure 7.** Mean of mean Hb of new donors with alternative strategy

This figure shows the mean of the individual mean Hb values in donors who would be eligible to donate under the alternative algorithm, but who were previously deferred under current eligibility criteria. Results are presented across a range of α-mean values (x-axis), for each α-outlier setting (panel columns), and stratified by sex (panel rows). Country-specific curves are shown in color according to the legend.

At more conservative α-mean values (left of x = 0), the newly eligible donors have higher mean Hb levels, as only donors with higher historical Hb levels would be accepted. As α-mean increases (moving right), donors with lower historical mean Hb levels are gradually included, resulting in lower average Hb among this group of newly accepted donors.

This analysis provides insight into the safety margin introduced by the algorithm: even with more lenient α-mean values, the mean Hb level of newly eligible donors remains above the deferral threshold, though the average Hb naturally decreases as the algorithm becomes more permissive.


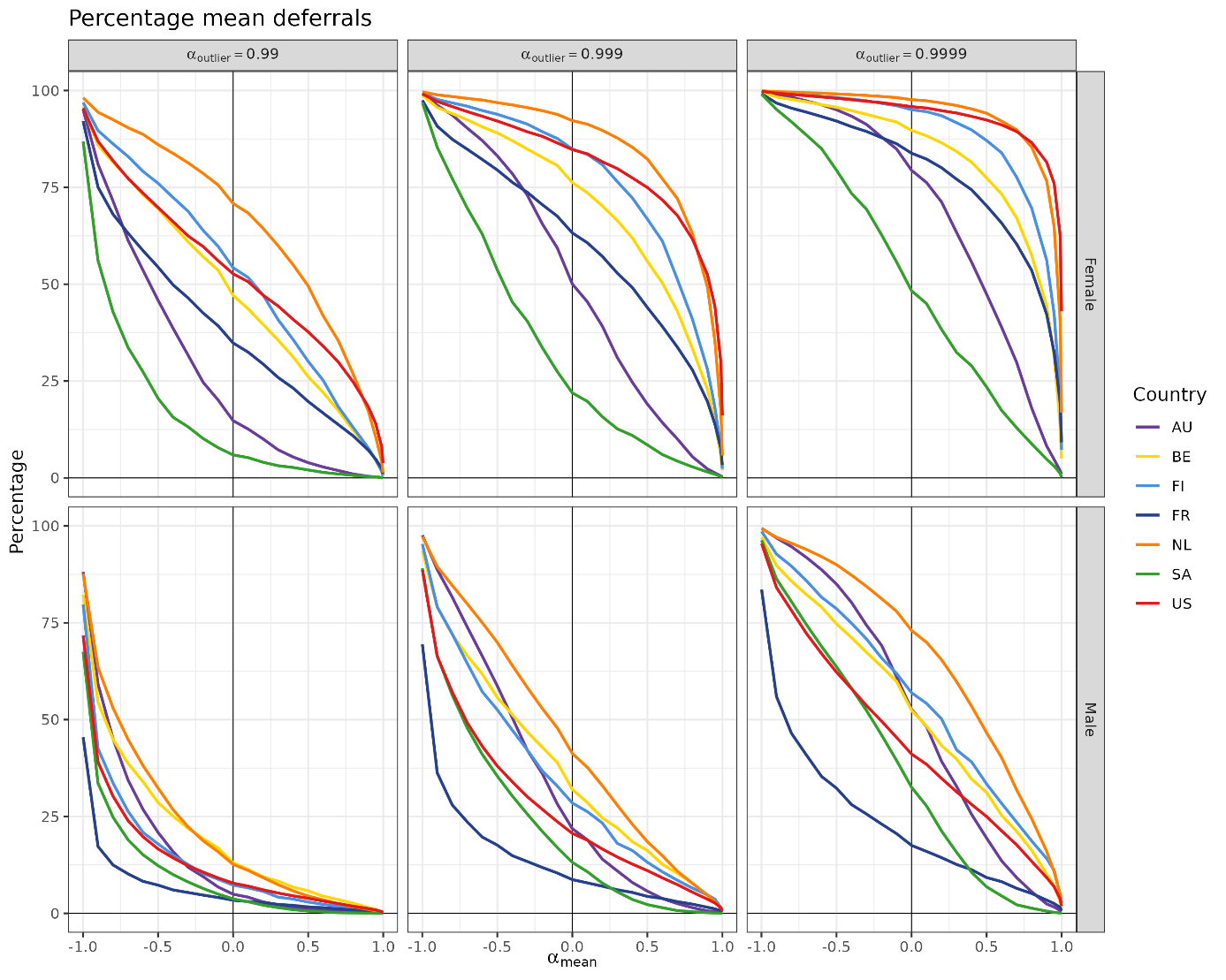


**Supplementary Figure 8.** Percentage of mean deferrals


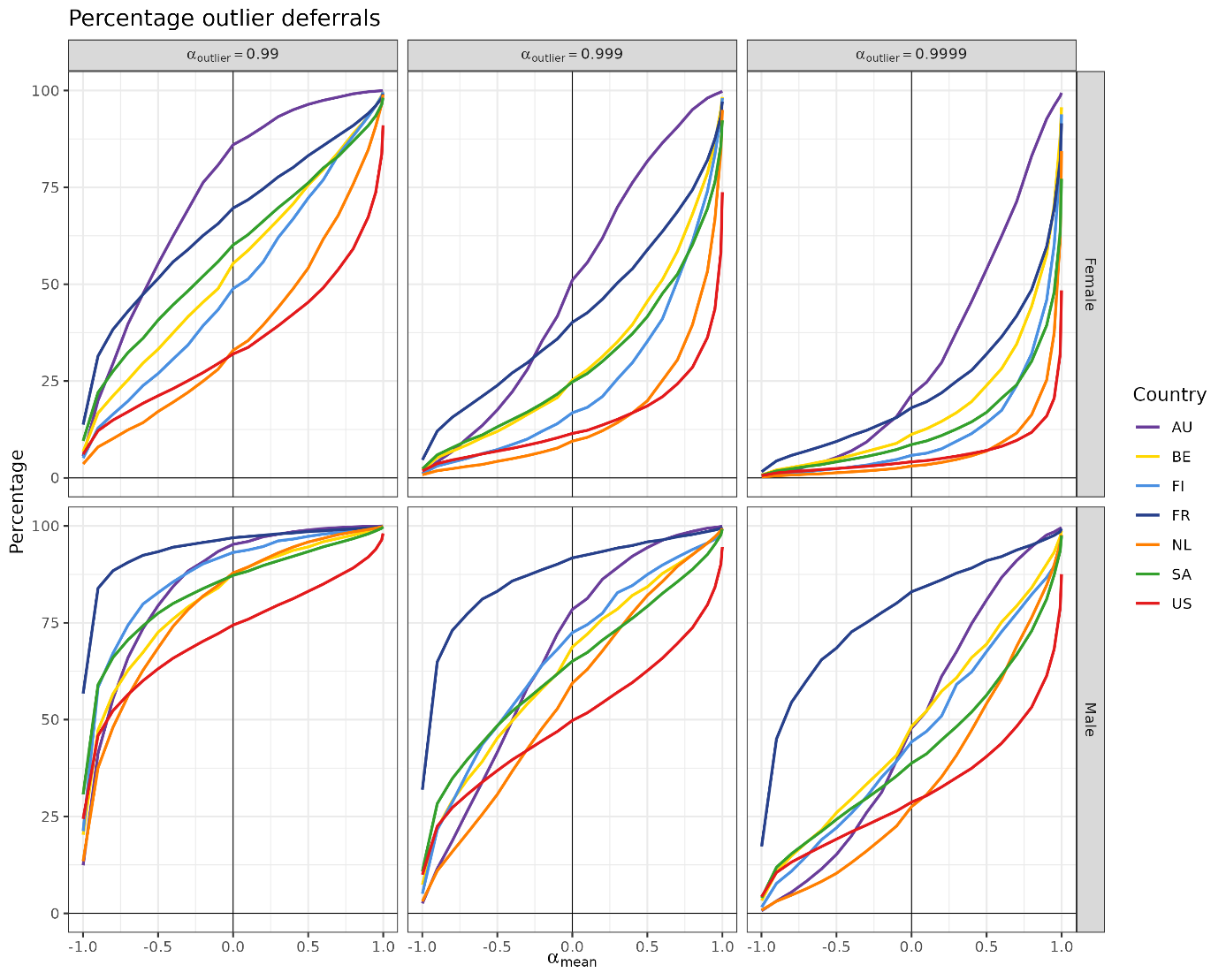


# **Supplementary Figure 9.** Percentage of outlier deferrals

Supplementary Figures 8 and 9 show the proportions of deferrals that are due to the α-mean (Figure 8) and α-outlier (Figure 9). Results are presented across a range of α-mean values (x-axis), for each α-outlier setting (panel columns), and stratified by sex (panel rows). Country-specific curves are shown in color according to the legend.

At very conservative α-mean values (left-hand side), nearly all deferrals are driven by low mean Hb values, resulting in percentages close to 100%. As α-mean increases (moving right), fewer donors are deferred based solely on their historical mean Hb level, and a larger proportion of deferrals would be triggered by current low Hb measurements flagged as outliers instead.

This figure highlights how the alternative algorithm shifts the balance between long-term (mean Hb-based) and short-term (outlier-based) deferrals as the parameters change, with country- and sex-specific differences reflecting variations in population Hb distributions and measurement variability.

# **Supplementary Table 1.** Sensitivity analysis results.

The analysis was performed with an α-mean of 0 and α-outlier of 0.999.

|  | **NL considering history of 4 donations for mean** | **USA min Hb** | **USA avg Hb** | **USA DRC excluded** |
| --- | --- | --- | --- | --- |
| **Donors** | 174,711 | 520,842 | 520,842 | 400,734 |
| **Donation attempts analyzed** | 2,097,670 | 3,546,947 | 3,547,014 | 2,626,121 |
| **Deferral rate with current strategy (%)** | 4.66 | 11.45 | 9.57 | 8.20 |
| **Deferral rate with alternative algorithm (%)** | 2.76 | 7.08 | 5.55 | 5.24 |
| **Change in deferrals (%)** | -40.8 | -38.2 | -42.0 | -36.1 |

These sensitivity analyses differ from the main analyses by limiting donor history to the last four donations in the Netherlands; and using the minimum Hb (from the repeated measurements), average Hb (from the repeated measurements), and excluding double red cell (DRC) donors in the USA.

For the analysis excluding DRC donors, we looked at DRC donations in the 12 months before the visit. For 4,293,990 (by 1,496,288 donors) of the 5,015,786 visits in the USA analysis we were able to establish whether there was a DRC donation. There were 100,583 unique donors (6.72% of donors) who made DRBC donations in that timeframe. When all visits of those donors were excluded, there were 3,853,386 visits to be analyzed.
